# Effect of trophic factor on single vascular insult induced amyloid accumulation with AD mice

**DOI:** 10.1002/alz70855_106567

**Published:** 2025-12-24

**Authors:** Mayank Pushpam, Rehab Hussain, Latha Diwakar

**Affiliations:** ^1^ Manipal Academy of Higher Education, Manipal, Karnataka, India; ^2^ Centre for Brain Research, Indian Institute of Science, Bengaluru, Karnataka, India

## Abstract

**Background:**

Alzheimer's disease (AD) is always presented as mixed dementia including vascular pathology. It is very often seen in elderly population that subjects with high vascular risk factors is associated white matter changes leading to cognitive decline. In case of AD such vascular changes are known to aggravate amyloid‐β (Aβ) plaques and tau deposits accelerating disease progression over age. In the present study we are interested in investigating vascular pathology such as blood brain barrier (BBB) changes and associated neuroinflammation in Aβ plaque formation leading to cognitive deficits. We are using AD transgenic mice to decipher molecular mechanisms which carries single or double mutations in APP protein and inducing vascular insults as well as protection by trophic factors.

**Method:**

ET‐1 (endothelin‐1 ‐ 2μg/2μl), a vasoconstricting peptide bilaterally injected into lateral ventricles of brain in AD mice. Behavioral parameters were performed in slow progressing APPswe mice after 30 days of ET‐1 injection. Immunohistochemistry was done in both APPswe and double mutant J20 AD mice to detect BBB structural changes for endothelial cells, junction and pericytes. Neuroinflammation and immune infiltration changes were measured with using specific markers. Pretreatment with pleiotrophin (PTN) was done to check protective effect on BBB.

**Result:**

Vasoconstriction caused by ET‐1 stimulates slow progressing APPswe mice for memory deficits even after 30 days of ET‐1 injection. There was BBB breaching seen as infiltration of macrophages probably triggering neuroinflammation. We also saw increased microglial activation in J20 as well as APPswe mice. This led to increased plaque deposition in J20 mice. The trophic factor PTN was able to prevent BBB disruption in APPswe mice.

**Conclusion:**

Vascular insult has varied response in slow progressing APPswe mice and fast progressing J20 mice in terms of susceptibility. This was reflected across age with increasing changes in BBB integrity. Pretreatment of trophic factor PTN was able to prevent BBB dysfunction caused by ET‐1 injection.

